# Natural and Biomimetic Antitumor Pyrazoles, A Perspective

**DOI:** 10.3390/molecules25061364

**Published:** 2020-03-17

**Authors:** Nádia E. Santos, Ana R.F. Carreira, Vera L. M. Silva, Susana Santos Braga

**Affiliations:** 1 LAQV-REQUIMTE, Department of Chemistry, University of Aveiro, 3810-193 Aveiro, Portugal; verasilva@ua.pt; 2 CICECO–Aveiro Institute of Materials, Department of Chemistry, University of Aveiro, 3810-193 Aveiro, Portugal; ritafutre@ua.pt

**Keywords:** pyrazole alkaloids, pyrazofurin, dystamycin analogues, curcuminoid pyrazoles

## Abstract

The present review presents an overview of antitumor pyrazoles of natural or bioinspired origins. Pyrazole compounds are relatively rare in nature, the first ones having been reported in 1966 and being essentially used as somniferous drugs. Cytotoxic pyrazoles of natural sources were first isolated in 1969, and a few others have been reported since then, most of them in the last decade. This paper presents a perspective on the current knowledge on antitumor natural pyrazoles, organized into two sections. The first focuses on the three known families of cytotoxic pyrazoles that were directly isolated from plants, for which the knowledge of the medicinal properties is in its infancy. The second section describes pyrazole derivatives of natural products, discussing their structure–activity relationships.

## 1. Introduction

**Figure 1:**
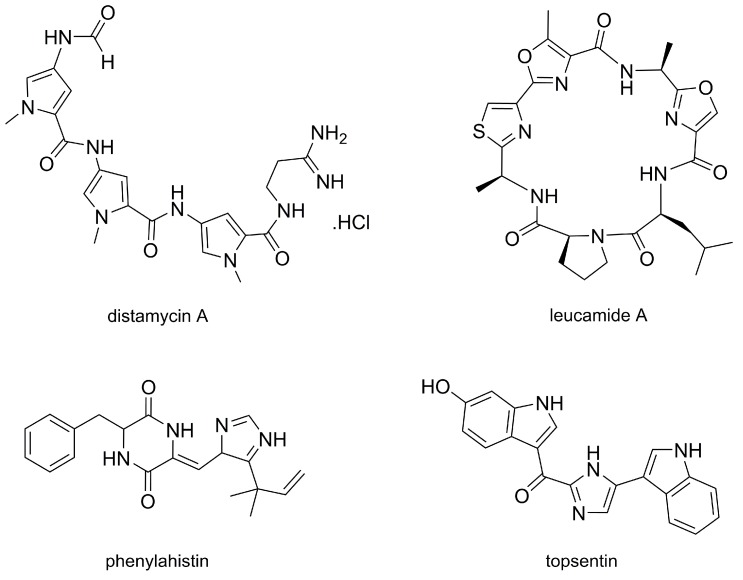
Structures of distamycin A, leucamide A, phenylahistin, and topsentin, four examples of natural antitumor compounds exhibiting a variety of pentameric heterocycles.

The biochemical machinery of plants and marine life is the source of an immense variety of compounds with medicinal activity. Natural products were, traditionally, the main source of medicinal actives, and today they continue to provide useful new drugs. Examples of natural antitumorals include paclitaxel (taxol) [1] and a few marine drugs such as cytarabine, eribulin, and trabectedin [2,3,4,5,6]. Notably, five-membered heterocycles are found in a variety of natural antitumor compounds. Figure 1 presents some examples of these molecules. The structural diversity of the pentameric heterocycles in such natural compounds is notable, from nitrogen heterocycles like pyrrole and imidazole, to pentameric cycles containing two kinds of heteroatoms like thiazole and oxazole. Distamycin A, obtained from *Streptomyces netropsis*, features several pyrrole rings; it is an antitumor, antimicrobial, and antiviral agent that acts by altering the conformation of DNA via irreversible binding to its A–T rich domains [7,8]. Leucamide A, isolated from the marine sponge *Leucetta microraphis* of the Great Barrier Reef of Australia, has an uncommon chemical structure with a mixed methyloxazole and thiazole pair. It has selective inhibitory action against tumor cells (being innocuous on microbes and algae) [9]. Phenylahistin is a metabolite of the fungus *Aspergillus ustus* that presents an imidazole moiety associated with a diketopiperazine [10]. It has antitumor activity via inhibition of tubulin polimerisation [11]. Topsentin is also a natural imidazole derivative. This compound is isolated from Caribbean deep-sea sponges of the *Spongosorites* genus, and it features antitumor activity on mouse models that has been associated with binding at the minor groove of DNA [12]. The promising properties of natural molecules such as topsentin, leucamide A, and dystamycin A have made them promising leads for the development of derivatives with improved activity (see, for instance, the family of compounds described in Section 3.2).

**Figure 2:**
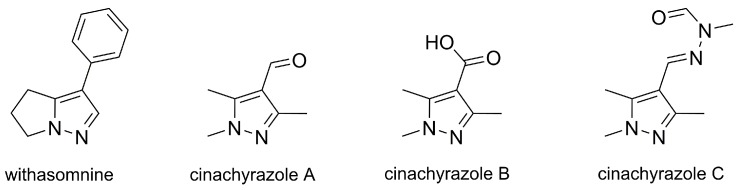
Structures of withasomnine and cinachyrazoles A, B, and C, pyrazole alkaloids occurring in plants and sea sponges.

Within pentameric heterocycles, pyrazoles, comprising two adjacent nitrogen atoms, are the less abundant ones in nature and also less known and explored as natural products. The scarcity of natural pyrazoles has been attributed to the difficulty in the formation of the N–N bond by living organisms [13]. Nevertheless, pyrazole is found in the structure of a few alkaloids, namely, withasomnine and cinachyrazoles A, B, and C (Figure 2). Withasomnine is a papaverin-like sedative that occurs in the roots of *Withania somnifera* [14], the root bark of *Newbouldia laevis* [15], and in *Elytraria acaulis* [16]. The cinachyrazoles A, B, and C are 1,3,5-trimethylpyrazole alkaloids recently isolated from sea sponge species of the genus *Cinachyrella*, and they have no known bioactivity, having displayed no cytotoxicity in tests with cultured cancer cell lines [17].

**Figure 3:**
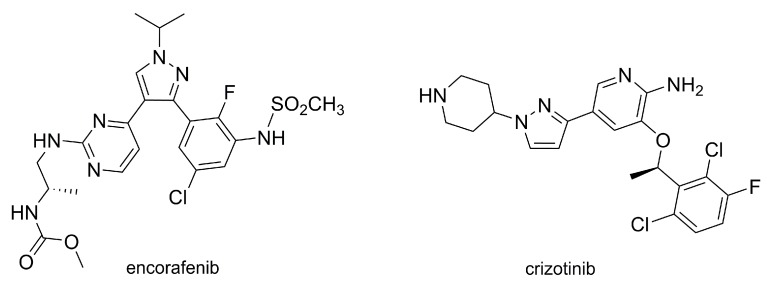
Antitumor pyrazoles available in the market: encorafenib (tradename Braftovi^®^) and crizotinib (tradename Xalkori^®^).

Notably, the pyrazole ring by itself (i.e., unsubstituted) was demonstrated to have some biological activity. It features anti-inflammatory properties in carrageenan-induced mice paw oedema [18], nephroprotecting action in cisplatin-induced mice kidney damage [19], and a caspase-mediated apoptotic effect on lung cancer cells (A549 line) [20]. The pyrazole core is also found in the structure of a few synthetic commercial drugs, such as the nonsteroidal anti-inflammatory agents celecoxib and lonazolac, and two antitumor drugs, represented in Figure 3: Encorafenib, for the oral treatment of skin cancer [21,22,23,24], and crizotinib [25,26,27], also taken orally and used in lung cancer.

This review is organized into two sections. It starts by presenting natural pyrazoles that have reported cytotoxic activity. The second section looks into the families of pyrazole compounds that derive from or are inspired by active natural molecules, identifying their main targets of action and comparing the activities of compounds within the same family to disclose some important structure–activity relationships. Data resulting from on their safety is presented when possible, and the implications towards a possible future medicinal use are discussed.

## 2. Antitumor Pyrazoles from Natural Sources

### 2.1. Pyrazole Alkaloids Found in Watermelon Seeds

**Figure 4:**
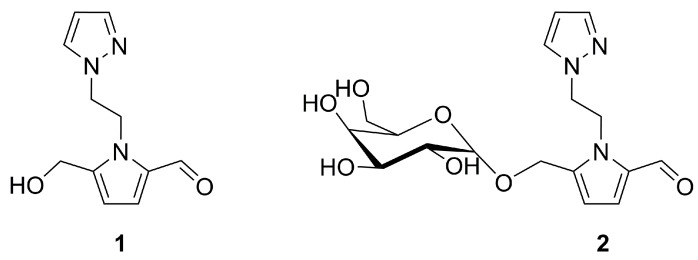
Structure of two pyrazole derivatives **1** and **2** isolated from the seeds of *Citrullus lanatus* watermelon. 1-[2-(5-hydroxymethyl-1*H*-pyrrole-2-carbaldehyde-1-yl)ethyl]-1*H*-pyrazole (**1**) showed no cytotoxicity, while 1-({[5-(α-d-galactopyranosyloxy)methyl]-1*H*-pyrrole-2-carbaldehyde-1-yl}- -ethyl)-1*H*-pyrazole (**2**) was mildly cytotoxic on mouse melanoma cells.

A new pyrrolopyrazole (**1**) and its galactosylated derivative (**2**), represented in the Figure 4, were recently isolated from the seeds of *Citrullus lanatus*, a species of watermelon [28]. These compounds were classified as pyrazole alkaloids and postulated to derive from l-α-amino-β-(pyrazolyl-*N*)-propanoic acid, a naturally occurring amino acid with a pyrazole ring, found in the juice of watermelons [13].

The cytotoxicity of compounds **1** and **2** was tested against a skin cancer cell line (mouse B16 melanoma 4A5 cell line). Mild activity was observed for compound **2**, with 70.4% growth inhibition at a concentration of 100 μM, but not for compound **1**. This can be attributed to the higher solubility and incorporation of **2** into the cells that results from the presence of a sugar moiety in its chemical structure.

### 2.2. Pyrazofurin, A Natural C-Nucleoside

**Figure 5:**
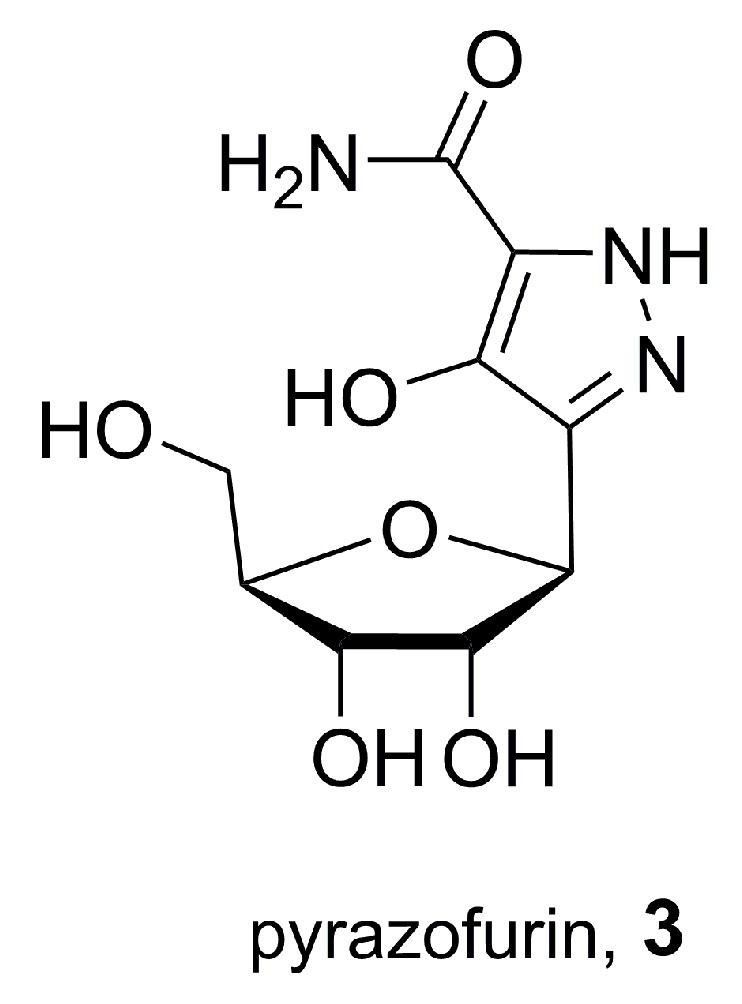
Pyrazofurin, or 3-β-d-ribofuranosyl-4-hydroxy-1*H*-pyrazole-5-carboxamide (**3**), a natural pyrazole obtained from *Streptomyces candidus* strains.

Pyrazofurin (**3**), depicted in Figure 5, is a naturally occurring nucleoside analogue produced by *Streptomyces candidus*. It has been known for decades, but only very recently has its biosynthesis begun to be elucidated, with researchers unveiling the involvement of PyrN, a new enzyme capable of forming the N–N bond [29]. Since its discovery in 1969, pyrazofurin has been the target of much interest due to its antimicrobial, antiviral, and antitumor properties, and for this reason a patent covering its bioindustrial production process was filed on the same year (and granted in 1974) [30].

Pyrazofurin works as an antimetabolite, inhibiting orotidine-5′-monophosphate decarboxylase and stopping the biosynthesis of pyrimidine [31]. Its antineoplastic activity was demonstrated in rats, and a relatively broad range of tumors were shown to be sensitive to it, including Walker carcinosarcoma, Ca755 adenocarcinoma, plasma cell myeloma, and various types of lymphosarcoma and of breast carcinoma [32]. Phase I clinical trials were conducted on human patients with disseminated cancer, but objective tumor regression was not observed in any of the 50 patients deemed suitable for response evaluation [33]. Following this study, interest in pyrazofurin as an antitumor drug has faded. Nevertheless, and considering the modern tools and methods available for chemical modification procedures, this molecule is worth revisiting as an inspiring model to design derivatives with better activity [34].

### 2.3. Pyrazole Derivatives from the Tall-stilted Mangrove Tree

The tall-stilted mangrove, *Rhizophora apiculata*, is a tree of the Indo-West Pacific that grows in intertidal wetlands in close association with other species of the mangrove. Used in traditional medicine, stilt mangrove bark is associated with treatment of angina, boils, and fungal infections; leaves and bark have been used as an antiseptic and to treat diarrhea, dysentery, fever, and malaria, albeit with unknown effectiveness rates [35].

**Figure 6:**
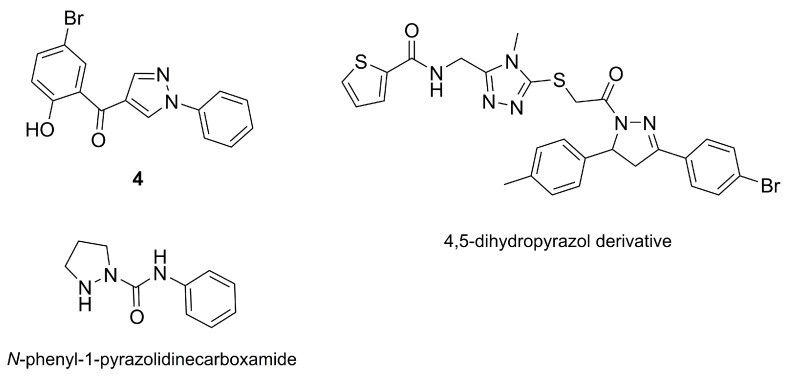
Structures of three selected compounds from those occuring in the methanol extract obtained from the whole plant of *R. apiculata*, which include a pyrazole derivative, (5-bromo-2-hydroxy-phenyl)-(1-phenyl-1*H*-pyrazol-4-yl)ketone (**4**), a dihydropyrazole derivative, and a pyrazolidine derivative.

Investigation of the main active components in *R. Apiculata* was conducted by preparing methanol extracts of the whole plant and analyzing their composition [36]. Characterization of the composition of the extract has revealed the presence of a new pyrazole derivative (**4**) as well as several other compounds, including a 4,5-dihydropyrazyltriazole derivative and *N*-phenyl-1-pyrazolidinecarboxamide, all depicted in the Figure 6. Moreover, the extract displayed tumor-reducing properties in mouse melanoma models, with reduction of tumor volume, increased body weight, and a 50% raise in the survival rate after 30 days. As a remark to the report of Prabhu et al. [36], we note that the antitumor activity reported for the extract cannot be directly correlated with compound **4**. There are multiple chemical entities in the extract, and the activity may result from one of them or from a combined effect. It would thus be interesting to isolate and evaluate the different compounds in the extract to allow for the identification of the active molecules and possible synergies between them.

## 3. Pyrazole Analogues of Natural Products

### 3.1. Curcuminoid Pyrazoles for Telomerase Inhibition

Curcumin, a diaryl heptano-3,5-dione, is the main active compound present in the rhizome of *Curcuma longa* (turmeric). Used for centuries as a traditional medicine, curcumin is able to act on multiple biological targets, thus having a varied set of activities: anti-inflammatory, antioxidant, and antitumor. The later has, in recent years, gained growing recognition due to the good results from clinical trials on patients with various types of cancer [37].

A known target of curcumin is telomerase. Curcumin interferes with the expression of the genes that encode hTERT, an RNA component of telomerase [38,39], thus increasing telomerase expression and activity. This enzyme has the function of repairing damage to the ends of the DNA caused by continuous replication, being active in stem cells and dormant in adult somatic cells. Reactivation of telomerase is a critical step in carcinogenesis, as it makes neoplasic cells immortal, that is, able to replicate indefinitely.

**Figure 7:**
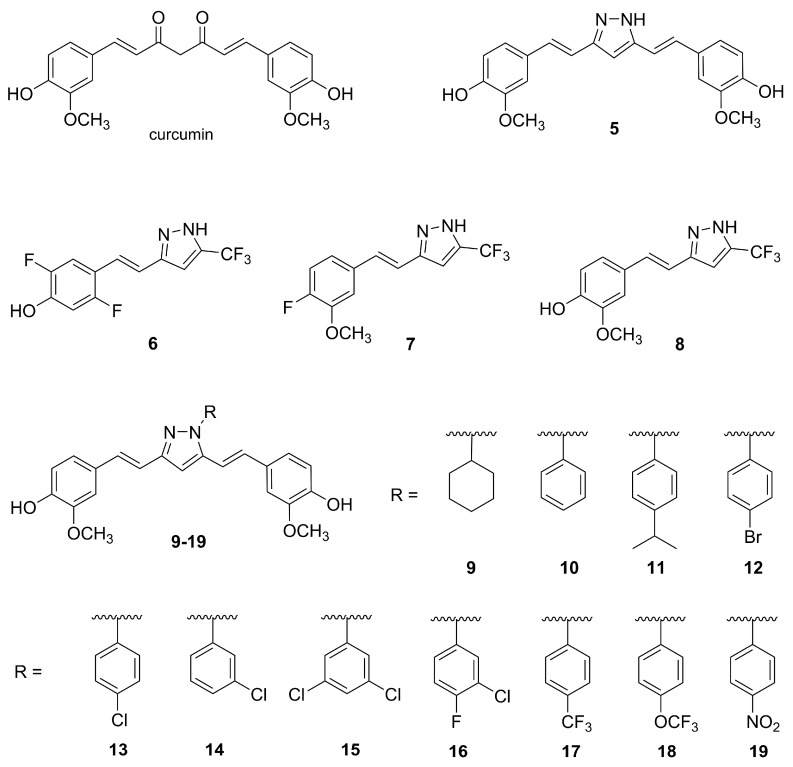
Structures of curcumin and of selected compounds from two families of curcuminoid pyrazoles: curcumin pyrazole, **5**; the hemicurcumin pyrazoles **6**–**8** [41]; and the 1*H*-substituted derivatives **9**–**19** [42].

Curcuminoid pyrazoles are a class of curcumin analogues obtained by replacement of the diketone moiety with a pyrazole ring. They were first developed as anti-inflammatory agents [40]. In recent years, with the discovery of hTERT as one of the targets of curcumin, these structures became interesting for cancer therapy. In a first set of studies, a library of thirteen curcuminoid pyrazoles was prepared and screened for cytotoxicity using the cancer cell lines HeLa (cervix carcinoma), HT-29 (colon carcinoma), and MCF-7 (breast cancer), and a nontumor line, HEK-293 (human embryonic kidney cells) [41]. The compounds **5**–**8**, represented in Figure 7, are highlighted in the present review for their superior performance. Notably, all the studied compounds were able to inhibit the growth of HeLa cells, with IC_50_ values ranging from 1.7 ± 0.6 μM for compound **5**, to 36.1 ± 0.4 μM for the less potent compound (not shown). Against the cell lines MCF-7 and HT-29, the most active compounds were **5** and **7**, with IC_50_ values under 15 μM. The compounds were further investigated regarding their ability to inhibit the expression of the hTERT gene using quantitative PCR (RT-qPCR). Compounds **5**, **6,** and **8** strongly downregulate hTERT gene expression to 49%, 42%, and 44%, respectively, of the value for nontreated cells. Note that these values are higher than the one shown by curcumin, which reduced hTERT expression to 59%. The safety of the compounds was evaluated on nontumor cells of embryonic kidney line HEK-293. The IC_50_ values against this cell line were quite low, which raises severe safety issues. The most striking case is that of compound **5**, more toxic on nontumor cell line than against the tumor lines. In fact, **5** has an IC_50_ of 0.72 ± 0.35 μM on the embryonic kidney HEK-293 cells. Compound **7** also performed poorly, with an IC_50_ of 2.9 ± 0.6 on this cell line. Overall, all the compounds are toxic to kidney cells as their IC_50_ values are below 55 μM.

Another family of curcuminoid pyrazoles was developed by inserting, in the 1*H*-position of the pyrazole, a variety of substituents, with higher predominance of aryl groups [42]. Their cytotoxic evaluation was conducted on the HeLa, MCF-7, and A549 (lung adenocarcinoma) cell lines. Figure 7 presents a selection of eleven compounds, **9**–**19**, that had the strongest activities. All of these eleven compounds had IC_50_ values lower than 20 μM against the three tumor cell lines tested. Regarding growth inhibition of cells of the MCF-7 line, compounds **11** and **12** were the most active ones, with IC_50_ values of 6.0 ± 0.7 and 5.8 ± 0.4 μM, respectively. Compound **12** also performed well against the A549 and HeLa cell lines, with IC_50_ values of 8.0 ± 1.4 and 9.8 ± 0.8 μM, respectively. Studies on the toxicological safety of these compounds on healthy cells were not conducted. Considering the abovementioned toxicity of compound **5** (the parent curcumin pyrazole), studies with nontumor cell lines are vital for the realistic assessment of the applicability of these compounds in tumor chemotherapy.

### 3.2. Pyrazole-bearing Dystamycin Analogues as DNA Alkylating Agents

**Figure 8:**
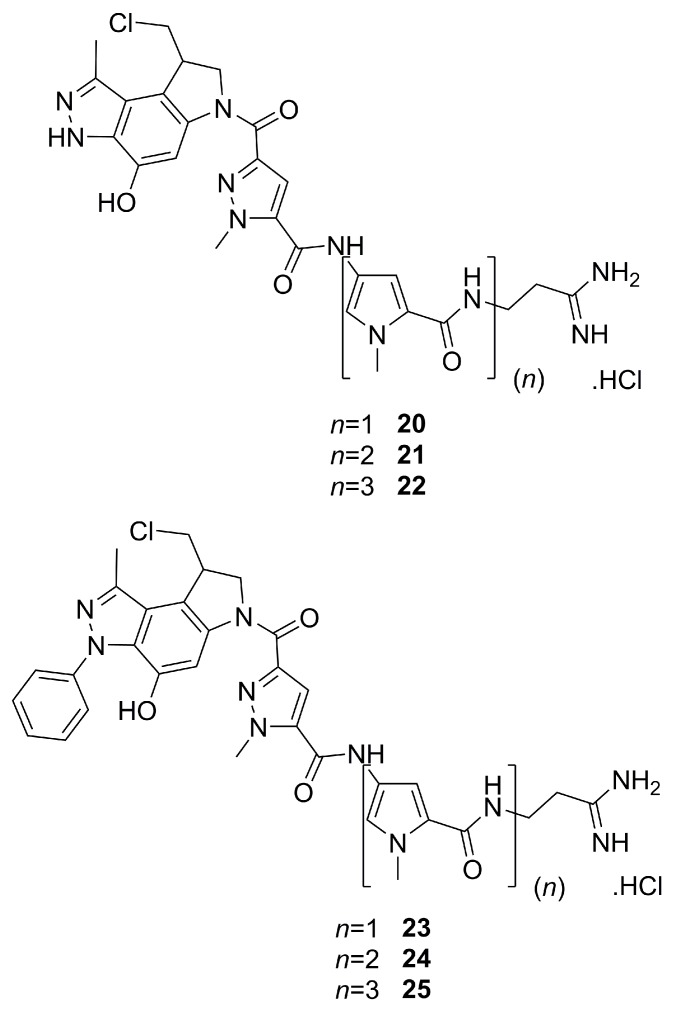
The six most active compounds from a family of pyrazole analogues of distamycin A with DNA alkylating activity [43].

Distamycin A has, as mentioned in the introduction, the ability to recognize and bind to DNA regions rich in A-T pairs, which means that it can be used as a vector for DNA. This concept was employed to create a new family of DNA alkylating agents. These compounds exhibited two active subunits: the first one, meant to act as a vector, comprised pyrazole analogues of distamycin A; the other one was a DNA-alkylating subunit [43]. Figure 8 shows a selection of the six most active compounds, **20**–**25**. They were evaluated for cytotoxicity using three human cell lines associated with blood cancer: T-lymphoblast (Molt/4 and CEM cell lines) and B-lymphoblast (Daudi cell line). In general, compounds **20**–**22** performed better than compounds **23**–**25**. A special highlight should be given to compound **22**, the most active of the entire family, featuring IC_50_ values in the nanomolar range (between 7.4 and 71 nM, depending on the cell line). Also noteworthy is the direct correlation between the increasing number of pyrrolic rings in the **20**–**22** series and the increase in antiproliferative activity. In turn, for the series of compounds **23**–**25**, the activity seemed to follow an inverse pattern, with compound **23**, having only one pyrrole ring, being the most active.

**Figure 9:**
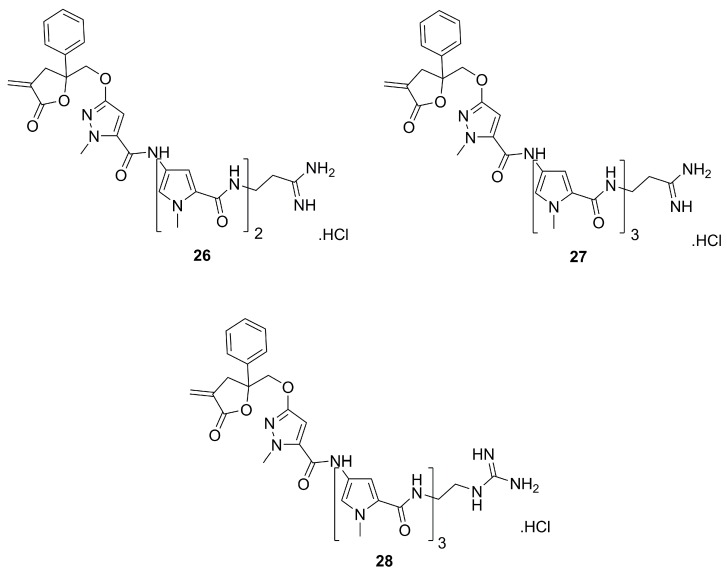
The three most active compounds from a second-generation family of pyrazole analogues of distamycin A with DNA alkylating properties and apoptosis-inducing activity [44].

A second family of pyrazole analogues of distamycin A agents comprised an α-methylidene-γ-butyrolactone as the alkylating subunit [44]. In this series, some compounds, such as **26**–**28** (Figure 9) had a phenyl substituent at the γ-position of the lactone, while a few others (not shown) presented a methyl group in the same position. The phenyl-containing compounds, **26**–**28**, were those with better activity against human lymphoma cell lines (Molt/4 and CEM8). They also exhibited apoptotic activity on the human leukemia cell line HL-60. Also noteworthy is the fact that the polypyrrolic frame in compounds **26**–**28** was lengthy (*n* = 2 or 3). Thus, compounds **26**–**28** are structurally very similar to dystamycin A, which has three pyrroles (*n* = 3). This may lay a structural rationale for their high cytotoxicity. On the other hand, compound **26**, with only two pyrroles, was the strongest in inducing apoptosis.

### 3.3. Phthalazinone-derived Pyrazoles for Aurora Kinase Inhibition

**Figure 10:**
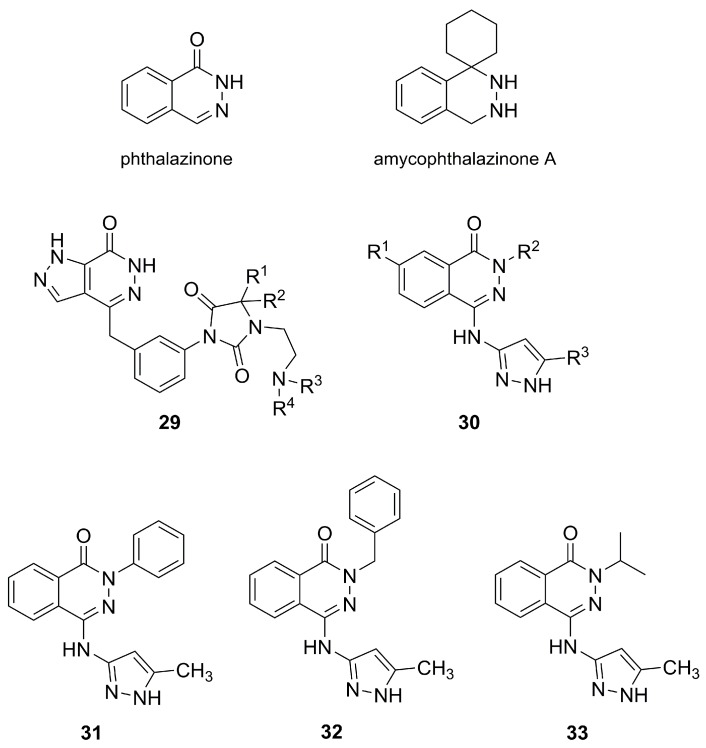
Phthalazinone, its natural derivative amycophthalazinone A, and its pyrazole derivatives: families **29** [47] and **30** [48] (with high structural diversity of substituents at positions R^1^, R^2^, R^3^, and R^4^), and compounds **31**–**33** [50].

The phthalazinone skeleton is an uncommon structure in natural compounds that seems to be associated with the metabolism of some fungal species. It is easily metabolized to phthalazine N-oxide by *Fusarium monoliforme* and *Cunninghamella elegans* [45]. A natural derivative of phthalazinone was isolated in 2019 from lichens of the *Amycolatopsis* genus [46]. This new natural compound was called amycophthalazinone A (Figure 10), and it has shown fair antimicrobial activity against *Staphylococcus aureus*, *Salmonella typhi*, and *Candida albicans*.

Phthalazinone is also an important pharmacophore for antitumor activity. Several derivatives, including the family of compounds **29**, in which the benzene ring of the structure of phthalazinone is replaced by pyrazole moiety, were patented for use as adjuvants in cancer chemotherapy [47]. In another patent, compounds of the family numbered generically as **30** were equally claimed for use as antitumor agents, either alone or in combination [48]. This family comprised a very large variety of pyrazolophthalazinones for which the antitumor activity was attributed to Aurora kinase inhibition [48,49]. Aurora kinases A and B are two enzymes involved in different steps of the cell division process. Their inactivation allows stopping the mitosis process and, subsequently, inhibiting tumor growth via apoptosis.

A small library of compounds, **31**-**33**, bearing a 5-methylpyrazole attached to the phthalazinone core (Figure 10), was developed and studied in comparison with compounds bearing a nonsubstituted pyrazole (not shown) [50]. Structure–activity relationship studies have demonstrated that the presence of the 5-methylpyrazole brought higher activity than nonsubstituted pyrazole, which was explained by the fact that the methyl group can easily bind in a largely hydrophobic pocket. Compounds **31**–**33** were excellent inhibitors of Aurora kinase A, with values in the nanomolar range (IC_50_ of 31, 65, and 35 nM, respectively); compound **31** was also able to inhibit Aurora kinase B, with an IC_50_ of 30 μM. In vitro cytotoxicity studies against MCF-7 breast cancer cell line showed IC_50_ values of 1.6 μM for **31** and **33** and of 12.6 μM for **32**.

A subsequent bioavailability evaluation in mice showed that all three compounds displayed plasma concentrations above the cellular IC_50_ at the half-life and adequate oral bioavailability. The half-life itself is relatively short, ranging between roughly two and four hours, and after 24 h the compounds were completely eliminated from the body [50].

## 4. Conclusions

Pyrazole compounds are seldom found in nature. They were first identified in natural products in the 1960s, which witnessed the discovery of withasomnine, a pyrazole alkaloid with sleep-inducing properties. In the 1970s, the first natural pyrazole with cytotoxic activity, pyrazofurin, was reported. Now, the number of known natural pyrazoles has increased, especially in the last decade, and more of these compounds are expected to be discovered in the forthcoming years as a result of the growing efforts in natural product identification. Very little is known, however, about the ability of these compounds to stop tumor growth. Compound **2** was only tested on one tumor line (melanoma), having displayed mild cytotoxicity. Regarding compound **4**, the activity still needs to be corroborated as it was tested in tandem with other compounds as part of a multicomponent plant extract.

The herein gathered reports of bioinspired pyrazoles show more promising outcomes, albeit some of these families of compounds remain a few steps away from translation into the clinic. Curcuminopyrazoles (**5**–**8**) have a good antitumor action, but their structures need further improvement to achieve good toxicological safety profiles. Pyrazole-bearing analogues of dystamycin A, carefully designed to bind irreversibly to DNA, are excellent in inducing apoptosis of leukemia cells (HL-60). However, studies on their safety regarding healthy cells are still unavailable. Finally, pyrazolophthalazinones are gaining recognition as an emerging class of selective antitumor drugs. Several of these compounds (families **29** and **30**) are already patented. New families under development include the one of compounds **31**–**33**. These combine very potent activity with adequate bioavailability and a short time of permanence in the body, which helps ensure reduced exposure and thus minimize any potential secondary effects.
